# Proposal for a new N-stage classification system for intrahepatic cholangiocarcinoma

**DOI:** 10.3389/fonc.2023.1149211

**Published:** 2023-08-11

**Authors:** Shan Liao, Ruizhe Liao, Huaxing Wu, Shijie Wang, Yanming Zhou

**Affiliations:** ^1^ Department of General Surgery, First Affiliated Hospital of Xiamen University, Xiamen, Fujian, China; ^2^ Department of General Surgery, Jinling Hospital, Medical School of Nanjing University, Nanjing, China

**Keywords:** hepatopancreaticobiliary surgery, intrahepatic cholangiocarcinoma, lymph nodes, surgery, survival

## Abstract

**Background:**

The number of metastatic lymph nodes (MLNs) is not considered in the nodal status (N classification) of intrahepatic cholangiocarcinoma (ICC) in the current 8^th^Edition of the American Joint Committee on Cancer (AJCC) staging system. The aim of this study was to find out the optimal cut-off point based on the number of MLNs and establish a modified AJCC staging system for ICC according to the new N category

**Methods:**

A total of 675 ICC patients diagnosed between 2004 and 2015 were retrieved from the Surveillance, Epidemiology and End Results (SEER) database. The optimal cut-off value of MLNs affecting survival was determined by X-tile software. The relative discriminative power was assessed by Harrell’s concordance index (C-index) and Akaike information criterion (AIC).

**Results:**

The proposed new nodal category subdivided patients into three groups (N0, no MLN; N1, 1–3 MLNs; and N2, ≥ 4 MLNs) with significantly different overall survival (*P* < 0.001). Multivariable analysis revealed that the new nodal category was an independent prognostic factor (*P* < 0.001). Both the C-index and AIC for our modified staging system were better than those for the 8^th^ AJCC edition (0.574 [95% confidence interval 0.533-0.615] *versus* 0.570 [95% confidence interval 0.527-0.613], and 853.30 *versus* 854.21, respectively).

**Conclusion:**

The modified AJCC staging system based on the number of MLNs may prove to be a useful alternative for predicting survival of ICC patients in clinical practice.

## Introduction

Intrahepatic cholangiocarcinoma (ICC) is the second most common malignant hepatic tumor, accounting for 10–15% of all primary liver cancers (1). Data have demonstrated that both the incidence and mortality of ICC are concomitantly increasing over the past few decades (2). Surgical resection remains the only potential therapy that can cure ICC patients (3, 4). However, only 20–40% of patients present with resectable disease at the time of diagnosis, and even in this subset of patients undergoing liver resection, recurrence is a common event with a poor 5- year survival of 30–35% (5). An accurate staging protocol is essential for prognostic stratification and determining treatment strategy.

The American Joint Committee on Cancer (AJCC) tumor-node-metastasis (TNM) staging system is the most regularly used predictive model for malignant tumors. The new 8^th^ edition staging system for ICC has made some major changes as compared with the 7^th^ Edition, including re-definition of the T and overall stage categories (6). The new T-staging system used a tumor size cutoff of 5 cm to separate the T1 category into T1a and T1b. Additionally, instead of indicating periductal invasion, T4 now is defined as involvement of local extrahepatic structures by direct invasion. However, a validation study has demonstrated that the 8^th^ Edition of AJCC staging system is no better than previous staging systems in providing prognostic relevance (7). In particular, the N classification has incurred criticism because it simply describes the lymph node status as node-negative (N0) and a node-positive (N1) (8). The number of metastatic lymph nodes (MLNs) has been identified as a significant prognostic factor in many other types of gastrointestinal carcinomas and is incorporated into their respective staging protocols (9–11).

Recently, Zhang et al. proposed a new N classification for ICC based on analysis of 15 universal high-volume centers and the Surveillance, Epidemiology and End Results (SEER) Registry as node-negative (N0), 1–2 metastatic lymph nodes (MLNs) (N1), and ≥ 3 MLNs (N2) (8). In the present study, we sought to optimize the cut-off values of the number of MLNs different from Zhang’s and more accurately stratify ICC patients by utilizing the SEER population database. Subsequently, we compared our proposed nodal sub-stages with those proposed by Zhang et al. Additionally, we attempted to modify the 8^th^ AJCC edition for ICC based on a new N staging system, hoping that it could help better determine the curability and prognosis of ICC patients by planning more appropriate multi-modality therapies.

## Methods

Consent to participate was waived as SEER data are publicly available.

### Patients and data collection

The study cohort of ICC patients who underwent surgical resection between 2004 and 2015 was enrolled from the SEER database based on the 3rd Edition of the International Classification (ICD–O–3) histology codes (8031, 8160, 8140, 8162, 8246, 8490) and the primary site code for intrahepatic bile duct (22.1). Patients meeting the following criteria were considered eligible for inclusion: (1) aged >18 years or older; (2) had at least one examined LN; and (3) histopathological diagnosis of ICC. Exclusion criteria were: (1) pathological diagnosis unknown; (2) follow-up information unknown; (3) tumor staging unknown; and (4) information of the number of MLNs unknown.

All patients identified in the current study were regrouped in the light of the 8^th^ AJCC Staging System on the basis of the existing information from the 6^th^ and 7^th^ editions of the AJCC Staging System. Except for the clinicopathological variables discussed above, the patient characteristics investigated in the test set from the SEER database should include gender, age, race, tumor size, code of extension, total number of positive LNs, tumor differentiation, survival time, and survival status. Using the extent of disease (EoD) and the collaborative staging (CS) provided by SEER to define the retrieved TNM information based on the following variables: AJCC stage group (6^th^ edition; 2004+), AJCC stage group (7^th^ edition; 2010+), CS Extension (2004–2015).

### Statistical analysis

The Kaplan–Meier method (log-rank test) was used to construct survival curves. Continuous variables are presented as median (range), and categorical variables are presented as frequency (percentage). Univariable Cox proportional hazard models were used to evaluate associations between the subgroups of metastatic LN counts and other variables, and all factors related to survival (*P* < 0.2) in univariable analysis were subjected to multivariable analysis. To find out the most significant cut-off points for discriminating overall survival (OS) in terms of the number of MLNs, we used the X-tile software (https://medicine.yale.edu/lab/rimm/research/software/) to calculate the optimal value after excluding the patients with M1 disease. The prognostic stratification ability of the cut-off points of the number of MLNs was compared by survival analysis. Considering that the AJCC guidelines recommend at least 6 LNs should be examined for complete nodal staging (6), survival analysis was repeated after excluding patients with <6 retrieved LNs.

The Pearson’s test was used to compare frequencies of categorical variables between groups. The discriminatory power was assessed using the Harrell’s concordance index (C-index) and Akaike’s Information Criterion (AIC), where a higher C-index or a lower AIC value indicates a greater discriminatory capacity of the staging scheme. Statistical analysis was conducted by using SPSS version 22.0 (IBM Corporation, Chicago, IL) and R version 3.6.2 (Bell Laboratories, Murray Hill, NJ).

## Results

### Study population

Altogether 675 eligible patients with ICC diagnosed through 2004 to 2015 were identified from the SEER database. The detailed demographics and clinical variables are summarized in **Table 1**. The median age of the patients in the cohort was 62 (range 15–86) years. The number of LNs harvested ranged between 1 and 36 with a median of 3 and the median number of MLNs was 0 (0–12). Over half (n= 431, 63.9%) of the patients were confirmed to have positive LNs, and roughly one third (n= 244, 36.1%) have negative LNs. Two hundred and four (20.3%) patients had at least 6 LNs harvested and 471 patients (69.7%) had less 6 LNs harvested. Over half (n= 400, 59.3%) of the patients died during a median follow-up period of 20 months. The median OS for all patients was 30 (95% confidence interval [CI] 26.4–33.6) months.

**Table 1:** Demographic details and clinical characteristics of patients with intrahepatic cholangiocarcinoma.

Variables	No. of patients (n=675)
Race	
White	544 (80.6%)
Others	130 (19.3%)
Unknown	1 (0.1%)
Sex	
Female	375 (55.6%)
Male	300 (44.4%)
Age, years	
≤ 60	283 (41.9%)
> 60	392 (58.1%)
Year of diagnosis	
2004-2010	258 (38.2%)
2011-2016	417(61.8%)
Tumor differentiation	
Well/moderate	448 (66.4%)
Poor/undifferentiated	227 (33.6%)
Tumor size, cm	
≤ 5	319 (47.3%)
> 5	350 (51.9%)
Unknown	6 (0.8%)
T classification	
T1-2	528 (78.2%)
T3-4	147 (21.8%)
M classification	
M0	629 (93.2%)
M1	46 (6.8%)
Radiation therapy	
No	561 (83.1%)
Yes	114 (16.9%)
Statement	
Alive	275 (40.7%)
Dead	400 (59.3%)

### Cut-off values for metastatic LNs

**Figure 1:**
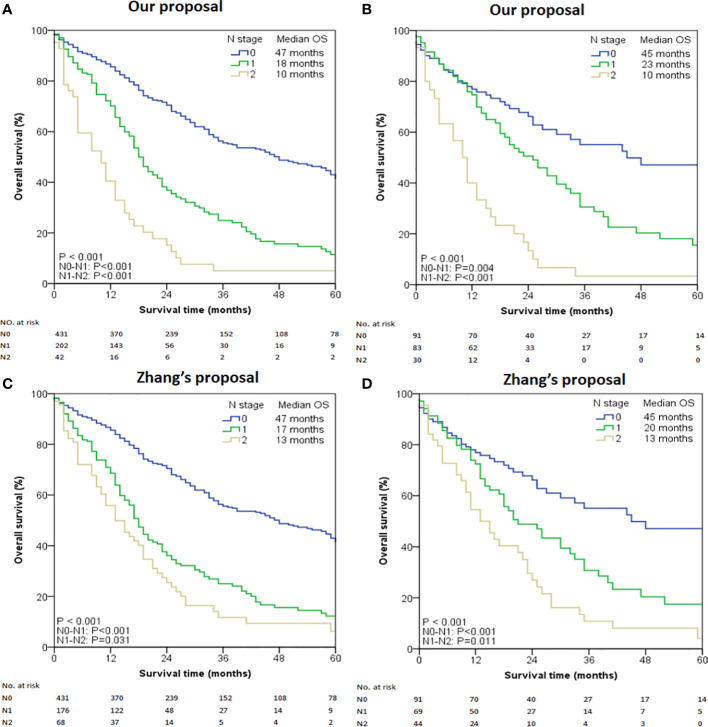
Kaplan-Meier curves stratified by the number of metastatic lymph nodes. **(A)** All cases using our proposed N stage. **(B)** Selected cases with LNs examined ≥ 6 using our proposed N stage. **(C)** All cases using Zhang’s proposed N stage. **(D)** Selected cases with LNs examined ≥ 6 using Zhang’s proposed N stage.

To gain the optimal set of the cut-off number of MLNs influencing survival, the X-tile program was used. Based on the result of survival data analysis with X-tile, the optimal cut-off points were revealed, based on which the group with 2 cut-off values (0 and 3) was finally chosen as the best set for survival discrimination in ICC patients. Using the proposed new N category, we further subdivided the patients into three groups: N0 (no MLN, n = 431), N1 (1-3 MLNs, n = 202), and N2 (≥ 4 MLNs, n = 42). The survival rate in patients with MLNs decreased rapidly and then gradually with the increased number of MLNs. The median survival time for N0, N1 and N2 was 47, 18 and 10 months, respectively (overall comparison, *P* < 0.001) (**Figure 1A**). Pair-wise comparisons further demonstrated significant differences between N0-N1 (*P* < 0.001), N1-N2 (*P* < 0.001), and N0-N2 (*P* < 0.001). The difference still existed in the subanalysis of patients with ≥ 6 retrieved LNs. The median survival time of the selected study cohort with N0, N1 and N2 diseases was 45, 23 and 10 months, respectively (overall comparison, *P* < 0.001) (**Figure 1B**). Pair-wise comparisons also showed significant differences between N0-N1 (*P* = 0.004), N1-N2 (*P* < 0.001), and N0-N2 (*P* < 0.001).

When the entire cohort was tested using Zhang’s proposed N stage, the median OS for N0, N1, and N2 was 47, 17, and 13 months respectively (overall comparison *P* < 0.001, **Figure 1C**). Pair-wise comparisons also demonstrated significant differences between N0-N1 (*P* < 0.001), N1-N2 (*P* = 0.031), and N0-N2 (*P* < 0.001). Similar results were also obtained in the cohort with ≥ 6 retrieved LNs (**Figure 1D**).

The discriminatory power of our proposed N staging system was consistently superior to that of Zhang’s proposal in terms of C-index (entire cohort: 0.524 [95% CI 0.491–0.557] *versu*s 0.515 [95% CI 0.482–0.548]; patients with ≥ 6 retrieved LNs cohort: 0.580 [95% CI 0.513–0.647] *versus* 0.576 [95% CI 0.505–0.647]) and AIC (entire cohort: 853.71 *versus* 860.89; patients with ≥6 retrieved LNs cohort: 236.92 *versus* 241.22). Additionally, the prognostic performance of our N staging system was also better than that of the 8^th^ Edition of AJCC Staging System (entire cohort: C-index 0.512 [95% CI 0.471–0.553], AIC 864.26; patients with ≥6 retrieved LNs cohort: C-index 0.561 [95% CI 0.485–0.637], AIC 248.70).

The correlation between the clinicopathologic variables and the new nodal category is shown in **Table 2**. Advanced (T3-T4) cancers were correlated with more positive LNs as compared with less invasive (T1-T2) cancers (*P* < 0.001). There was a clinically significant difference in the fact that distant metastases had more LN involvement (*P* < 0.001).

**Table 2:** Correlation between the clinicopathologic variables and the proposed new nodal staging.

Variables	N0	N1	N2	*P* value
Age, years				0.415
≤ 60	173 (40.0)	90 (44.6)	20 (47.6)	
> 60	259 (60.0)	112 (55.4)	22 (52.4)	
Sex				0.232
Female	243 (56.4)	114 (56.4)	18 (42.9)	
Male	188 (43.6)	88 (43.6)	24 (57.1)	
Tumor differentiation				0.283
Well/moderate	60 (66.0)	53 (63.9)	15 (50.0)	
Poor/undifferentiated	31 (34.0)	30 (36.1)	15 (50.0)	
Tumor size, cm				0.675
≤ 5	209 (48.8)	92 (46.2)	18 (42.9)	
> 5	219 (51.2)	107 (53.8)	24 (57.1)	
T classification				<0.001
T1-T2	364 (84.1)	139 (68.8)	25 (59.5)	
T3-T4	67 (15.5)	63 (31.2)	17 (40.5)	
M classification				<0.001
M0	415 (96.3)	181 (89.6)	33 (78.6)	
M1	16 (3.7)	21 (10.4)	9 (21.4)	

### Predictors of survival

Significant variables in univariable analysis were subjected to multivariable analysis, and results showed that the new nodal category, sex, age, tumor differentiation, T and M classification were independent prognostic factors (**Table 3**).

**Table 3:** Predictors of survival.

Variables	MST	Univariate analysis			Multivariable analysis		
		HR	95% CI	*P value*	HR	95% CI	*P value*
Race				0.298			
White	30	Reference					
Other	25	1.139	0.891-1.456				
Sex				0.017			0.060
Female	33	Reference			Reference		
Male	25	1.270	1.044-1.546		1.212	0.992-1.480	
Age, years				0.004			0.004
≤ 60	32	Reference			Reference		
> 60	28	1.217	0.997-1.486		1.355	1.102-1.665	
Year of diagnosis				0.039			0.038
2004-2010	25	Reference			Reference		
2011-2016	32	0.831	0.656-0.989		0.803	0.653-0.989	
Tumor differentiation				< 0.001			0.002
Well-moderate	36	Reference			Reference		
Poor-undifferentiated	19	1.568	1.280-1.922		1.391	1.129-1.713	
Tumor size, cm				0.192			
≤ 5	32	Reference					
> 5	25	1.141	0.936-1.391				
T classification				< 0.001			0.009
T1-T2	33	Reference			Reference		
T3-T4	18	1.730	1.382-2.166		1.366	1.079-1.728	
M classification				< 0.001			0.007
M0	32	Reference			Reference		
M1	14	1.994	1.420-2.798		1.609	1.137-2.277	
Harvested lymph nodes				0.003			0.867
1-5	32	Reference			Reference		
≥ 6	24	1.369	1.113-1.684		0.981	0.784-1.227	
New nodal category				< 0.001			< 0.001
N0	47	Reference			Reference		
N1	18	2.370	1.914-2.935		2.282	1.818-2.863	
N2	10	4.657	3.304-6.562		3.571	2.442-5.224	

MST, median survival time (months); HR, hazard ratio; CI, confidence interval.

### The proposed new TNM staging system

**Figure 2:**
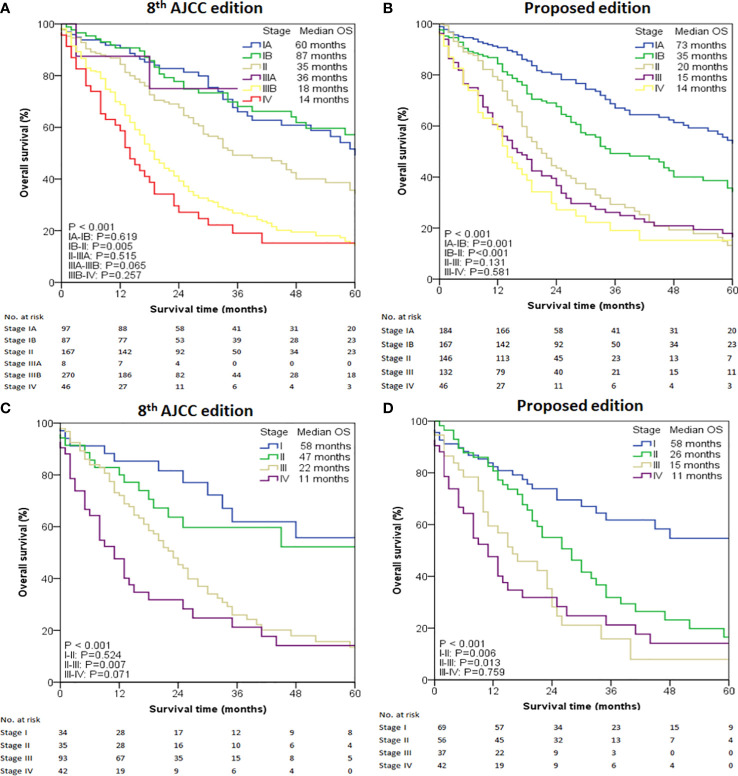
Overall survival (OS) analysis of the intrahepatic cholangiocarcinoma cases from the SEER database. **(A)** OS of all cases using the 8th edition of AJCC staging system. **(B)** OS of all cases using the modified staging system. **(C)** OS of selected cases with LNs examined ≥ 6 using the 8th edition of AJCC staging system. **(D)** OS of selected cases with LNs examined ≥ 6 using the modified staging system.

To predict the prognosis of ICC patients more accurately, we proposed a new TNM staging system based on the new nodal category described above (**Table 4**). This new TNM staging system retained the T and M definitions of the 8^th^ edition AJCC system of ICC. According to the 8^th^ Edition AJCC, the median OS of patients with IA, IB, II, IIIA, IIIB and IV was 60, 87, 35, 36,18 and 14 months, respectively in the entire study population (*P* < 0.001) (**Figure 2A**). Only pair-wise comparison of stage IB versus stage II showed significant prognostic difference (P = 0.032). Our new proposed staging system showed that the median OS of patients with IA, IB, II, III and IV was 73, 35, 20,15 and 14 months, respectively (*P* < 0.001) (**Figure 2B**), showing significant prognostic differences between stage IA and stage IB patients (*P* = 0.001) or between stage IB and stage II patients (*P* < 0.001)

**Table 4:** The AJCC staging definitions, and the proposed staging definitions for intrahepatic cholangiocarcinoma, With CrossTabulation of Stage Distributions.

AJCC eighth stage classification					Proposed stage classification				
T1a	solitary tumor ≤5 cm without vascular invasion				T1a	solitary tumor ≤5 cm without vascular invasion			
T1b	solitary tumor >5 cm without vascular invasion				T1b	solitary tumor >5 cm without vascular invasion			
T2	solitary tumor with intrahepatic vascular invasion or multiple tumors, with or without vascular invasion				T2	solitary tumor with intrahepatic vascular invasion or multiple tumors, with or without vascular invasion			
T3	tumor perforating the visceral peritoneum				T3	tumor perforating the visceral peritoneum			
T4	tumor involving local extrahepatic structures by direct invasion				T4	tumor involving local extrahepatic structures by direct invasion			
N0	no regional lymph node metastasis				N0	no regional lymph node metastasis			
N1	regional lymph node metastasis present				N1	1-3 regional lymph nodes metastasis present			
					N2	≥4 regional lymph nodes metastasis present			
M0	no distant metastasis				M0	no distant metastasis			
M1	distant metastasis				M1	distant metastasis			
AJCC eighth stage					Proposed stage				
Stage		T	N	M	Stage		T	N	M
IA		T1a	N0	M0	IA		T1	N0	M0
IB		T1b	N0	M0	IB		T2	N0	M0
II		T2	N0	M0	II		T3	N0	M0
							T1-3	N1	M0
IIIA		T3	N0	M0	III		Any T	N2	M0
IIIB		T4	Any N	M0			T4	Any N	M0
IV		Any T	Any N	M1	IV		Any T	Any N	M1
					AJCC eighth stage				
Systems					I		II	III	IV
Proposed stage				I	184		167	0	0
				II	0		167	146	0
				III	0		0	132	0
				IV	0		0	0	46

Given the low number of patients with ≥ 6 examined LNs, this cohort was classified as four stages. The median OS determined by the 8^th^ Edition AJCC was 58 months for stage I, 47 months for stage II, 22 months for stage III, and 11 months for stage IV (*P* < 0.001) (**Figure 2C**). Prognostic difference was observed only between stage II and III (*P* = 0.007) but not between stage I and II (*P* = 0.524) or between stage III and IV (*P* = 0.071). When the new proposed staging system was applied, the median OS of patients with I, II, III and IV was 58, 26, 15 and 11 months, respectively (*P* < 0.001, **Figure 2D**), showing statistically significant prognostic differences between all stages (*P* < 0.05) except for between III and IV (*P* =0.759).

In the entire cohort, both C-index and AIC for our modified staging system were better than those for the 8^th^ Edition AJCC (0.574 [95% CI 0.533-0.615] *versus* 0.570 [95% CI 0.527-0.613], and 853.30 *versus* 854.21, respectively). In patients with ≥6 retrieved LNs, C-index and AIC in our modified staging system were 0.632 (95% CI 0.556–0.708) and 250.81 respectively, both of which were also better than the corresponding values in the 8^th^ Edition AJCC (C-index 0.622 [95% CI 0.549–0.695], AIC 251.40).

## Discussion

The current AJCC staging system for ICC simply categorizes LNs as absent or present without considering the number of MLNs, despite many updates in sub-staging of the N stage for other carcinomas such as pancreatic adenocarcinoma, distal cholangiocarcinoma and gastric adenocarcinomas (10–12). This may be because only a limited number of studies have evaluated the survival outcome based on the number of MLNs in ICC. In 2005, a single-institution study involving 53 ICC patients demonstrated that 3 or more MLNs were associated with a worse prognosis (13). In a recent multi-institutional study from15 high-volume centers worldwide, Zhang et al. stated that subdivision of nodal disease into three categories (0, 1-2, or ≥ 3 positive nodes) had significant prognostic implications (8). However, the cutoff values were selected arbitrarily and the authors did not incorporate their new N-stages into the staging system. In contrast, we identified each possible cut-off value of the number of MLNs systematically by the X-tile program, and found that the number of MLNs had significant impact on the survival of ICC patients. More importantly, our further analysis showed that the overall discrimination of the proposed N stage system was superior to that of Zhang’s and the 8^th^ Edition of AJCC staging system. Although the current thresholds of N0 (no MLN), N1 (1-3 MLNs), and N 2 (≥ 4 MLNs) were identified to be consistent with other biliary malignancies such as perihilar cholangiocarcinoma and gallbladder cancer (8), the findings from the current study suggest that they are statistically sound. The analysis has unequivocally demonstrated that patients in group 1, 2 and 3 were all prognostically well differentiated.

While there is no unanimous answer to whether routine lymph node dissection (LND) should be performed in patients with ICC (14), we recommend that LND should at least be considered in that adequate LND can not only prolong survival but gain a better stratification of ICC patients. Although there are inadequate data to support our opinion discussed above, the fact that LN involvement is a prominent prognostic factor in ICC has already been confirmed in numerous other studies (15–18). It is common knowledge that an insufficient count of LNs retrieved may incur an increased risk of under-staging, especially in colorectal cancer and ampullary adenocarcinoma, knowing that their nodal stages have been recommended to harvest a minimum of 12 LNs (19, 20). Even though some recent studies (8) and the 8^th^ edition AJCC system of ICC (6) recommend harvesting at least 6 LNs to complete nodal staging, the other standpoints remain hotly debated. Nevertheless, a recent study has demonstrated that dissection of at least 5 LNs is required for ICC (21). However, the data source of this study is relatively single and small. In addition, compared with eastern Asian countries and regions, there is a low trend of routine LND in western countries (22), probably because Westerners have more fatty tissues, making LND more troublesome. Additionally, a routine LN gross examination showed that the more fatty tissues, the fewer the LNs could be found, because LNs can only be detected by sight and touch. Therefore, taking these into consideration, besides the total 675 cases of ICC after surgery from the SEER database to be analyzed, 204 patients with the examination of ≥ 6 LNs were included to further determine the potential optimal cut-off value of the number of MLNs in affecting the prognosis. As the median number of LNs collected for the ICC patients was 9 in the selected cases, the data from a minimum of 6 LNs examined may possibly be highly significant. For adequate LND (i.e., ≥ LNs), dissection of all fibroadipose and lymphatic tissue within the hepatoduodenal ligament between the hilar plate and the head of the pancreas, should be performed during hepatectomy in patients with ICC according to the AJCC guidelines (23). Specifically, dissection of no. 12 (hepatoduodenal ligament) and 8 (common hepatic artery) nodes is mandatory for accurate staging because more than 80% of MLNs manifested in these areas (24).

To the best of our knowledge, no study has reported the creation of a new staging system for ICC since the advent of the 8^th^ AJCC staging system. Here, we made a modification for ICC based on the new N stage system (**Table 4**) and obtained a better prognostic prediction compared to the 8^th^ AJCC edition.

Several reports in ICC and other malignancies have suggested that survival after surgery is correlated with the relationship of metastatic-to-examined LN, as evaluated by the LN ratio and log odds of MLNs (16, 25–28). However, the main obstacle in the assessment of these LN protocols is the lack of standard cut-off values for the risk stratifications. In fact, most cancer types have adopted the number of MLNs as N classification in the AJCC staging system (9–12).

The main limitation of this study is that we did not carry out perioperative subgroup analysis on the stratification of the resection margin status because we failed to find relevant information in the population-based SEER database. Similarly, the SEER does not report data on recurrent disease; as such, the impact of number of MLNs on timing and patterns of recurrence could not be assessed. Additionally, we could not analyze the statistical differences in patients with a limited number of pathologically examined LNs (< 6) because of the small number of N2 stage patients (29, 30).

## Conclusion

In summary, the current study demonstrated that patients with more positive LNs had poorer survival. According to this observation, we recommend to modify the current N classification of ICC into a 3-tier staging system on the basis of the number of MLNs: N0 (no MLN), N1 (1-3 MLNs), and N2 (≥4 MLNs). Besides, compared with the existing ICC staging systems, the new staging system described herein can obtain more accurate risk stratification, which could be considered for inclusion in the next version of the AJCC staging system.

## Author contributions

SL: methodology, formal analysis and writing—original draft. RL: funding acquisition, supervision. HW: formal analysis and writing—original draft. SW: data curation. YZ: conceptualization, supervision, funding acquisition, investigation, and methodology. All authors contributed to the article and approved the submitted version.
